# Gas Diffusion Electrodes Manufactured by Casting Evaluation as Air Cathodes for Microbial Fuel Cells (MFC)

**DOI:** 10.3390/ma9070601

**Published:** 2016-07-21

**Authors:** Sandipam Srikanth, Deepak Pant, Xochitl Dominguez-Benetton, Inge Genné, Karolien Vanbroekhoven, Philippe Vermeiren, Yolanda Alvarez-Gallego

**Affiliations:** Separation and Conversion Technology, Flemish Institute for Technological Research (VITO), Boeretang 200, MOL B-2400, Belgium; srikanth.sandipam@gmail.com (S.S.); deepak.pant@vito.be (D.P.); xochitl.dominguez@vito.be (X.D.-B.); inge.genne@vito.be (I.G.); karolien.vanbroekhoven@vito.be (K.V.); vermeirenphilippe@gmail.com (P.V.)

**Keywords:** gas diffusion electrode (GDE), bioelectrochemical systems (BES), microbial fuel cells (MFC), oxygen reduction reaction (ORR), low cost electrodes

## Abstract

One of the most intriguing renewable energy production methods being explored currently is electrical power generation by microbial fuel cells (MFCs). However, to make MFC technology economically feasible, cost efficient electrode manufacturing processes need to be proposed and demonstrated. In this context, VITO has developed an innovative electrode manufacturing process based on film casting and phase inversion. The screening and selection process of electrode compositions was done based on physicochemical properties of the active layer, which in turn maintained a close relation with their composition A dual hydrophilic-hydrophobic character in the active layer was achieved with values of ε_hydrophilic_ up to 10% while ε_TOTAL_ remained in the range 65 wt % to 75 wt %. Eventually, selected electrodes were tested as air cathodes for MFC in half cell and full cell modes. Reduction currents, up to −0.14 mA·cm^2−^ at −100 mV (vs. Ag/AgCl) were reached in long term experiments in the cathode half-cell. In full MFC, a maximum power density of 380 mW·m^−2^ was observed at 100 Ω external load.

## 1. Introduction

Microbial fuel cells (MFCs) are devices that produce electricity by means of oxidizing organic matter using microorganisms as catalyst at the anode. The electrons are transferred from the surface of a biocatalyst to the anode and then to the cathode through an external load, while, the ions (protons) migrate through an ion conducting or ion permeable membrane, or through the electrolyte between the electrodes. At the cathode, these electrons are accepted by an electron acceptor (mostly oxygen) in presence of the reducing equivalents (e.g., protons) to produce an electro-reduced substance (e.g., water, hydrogen peroxide) [1,2].

Similar to the anode, cathodes also play a very crucial role in enhancing the power output of MFCs by regulating the terminal reduction reaction. Various types of cathodes have already been tested for application in MFC, such as wet proofed carbon cloth, carbon fibers, graphite granules, carbon paper, Pt, Au covered Cu wires, graphite felt, graphite rod, graphite foam, graphite plate, carbon felt, activated charcoal, drilled graphite discs, reticulated vitreous carbon (RVC), etc. [3,4,5,6,7,8]. On the other hand, a variety of chemo/bio/electro catalysts also have been investigated as alternative cathodic catalysts in air-cathode MFCs [9,10]. Interestingly, uncatalyzed activated carbon has shown promising performance for the oxygen-reduction reaction (ORR) when compared to Pt [11]. Since low solubility of oxygen in aqueous solutions is one of the main problems, limiting the ORR by mass transfer, the use of gas diffusion electrodes (GDE) appears a promising option. GDE have large reaction areas and allow for higher mass transfer rates [12].

The development of porous gas diffusion electrodes (GDEs) for electrochemical cells containing a free electrolyte liquid phase typically relies on complex manufacturing techniques based on pressing, cold rolling and stepwise assembly of the different layers [13,14]. In order to make microbial electrochemical systems (MES) feasible in large scale applications, further work should aim at increasing not only the immediate efficiency of the biofuel cell electrode (by improving the chemistry), but also to consider the engineering problems [5]. It is imperative to produce the electrodes in a cheap manner and in sizeable dimensions instead of using costly materials and batch wise fabrication processes [15,16]. VITO has developed an electrode manufacturing process based on film casting and phase inversion [17,18]. The film casting technique makes it possible to produce large multilayered electrodes in a single run with a continuous manufacturing process. An important issue is that this film casting technique allows for different manufacturing steps to be varied in order to adapt the structure and the composition of the cast layers to any specific requirement, mostly depending on the application [19].

The most relevant variables are the characteristics of the active component (chemical composition, particle size, surface area, etc.), the characteristics of the polymer binder, and the ratio of active component to polymer binder. Other essential parameters are the type and the amount of solvent in the casting dope, the type of non-solvent in the extraction bath, the use of additional pore forming agents, the casting thickness and finally the extraction temperature [20,21]. The influence of several of these manufacturing parameters has already been studied and described in detail for mixed matrix organic-inorganic membranes based on ZrO_2_ and polysulfone [22,23].

In the study presented here, we have endeavored to produce air diffusion cathodes implementing the casting and phase inversion manufacturing method, and to evaluate their performance for application in MFC. The electrodes studied in this work consist of three layers, viz., a current collector, an electrochemically active layer (AL), and a hydrophobic gas diffusion layer (HGDL). The electrochemical reduction of O_2_ from air is performed in the AL. Its porosity is therefore of major importance. Furthermore, one should make a clear distinction between hydrophobic porosity and hydrophilic porosity. Indeed hydrophilic pores are required for the absorption of the electrolyte (e.g., wastewater) and the hydrophobic porosity is needed for the transport of air (O_2_) to the active sites of the carbon. The air is supplied to this AL through the HGDL, which also serves to avoid leakages, commonly referred to as weeping of the electrode [24,25,26,27].

## 2. Results and Discussion

### 2.1. Physico-Chemical Characterization of the Active Layer (AL)

**Figure 1:**
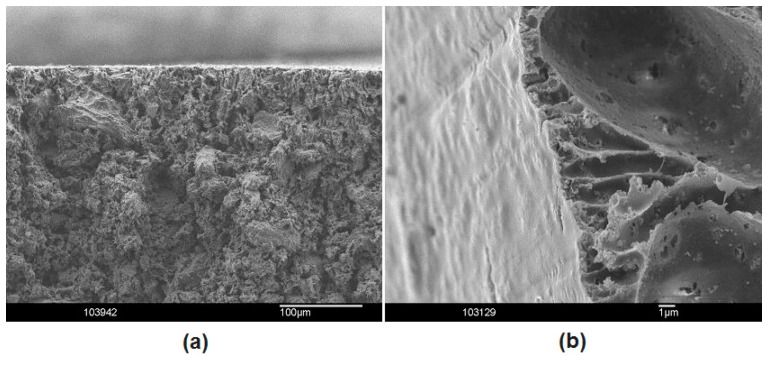
SEM image of the cross-section of a cast AL (**a**) and a cast HGDL (**b**).

The physicochemical properties of different types of AL were examined and compared to select the most promising electrode compositions for evaluation in a real MFC environment. The structure of the typical cross-section of an AL (75 wt % C–25 wt % PSf) and a HGDL (75 wt % FEP–25 wt % PSf) manufactured by casting are shown in Figure 1, left and right respectively.

#### 2.1.1. Influence of Fabrication Method on the Properties of the Active Layer

The properties of ALs with the same ratio active carbon to polymer, but based on different fabrication methods are presented in Table 1. It is noteworthy that varying the manufacturing technique implies changing the polymer binder: whereas PTFE is the polymer binder for VITO CoRE™ electrodes, PSf is the binder used for VITO CaSE™ electrodes. Both ALs compared in Table 1 were composed of 80 wt % of active carbon (Norit^®^SX1G) and 20 wt % of the respective polymer. A surprising observation is that, although it has the lowest available porosity (ε_TOTAL_), the VITO CoRE™ AL (based on PTFE as a binder) absorbs more water than the VITO CaSE™ AL. Indeed, since PTFE is the polymer with the lowest surface energy, it was expected that the PTFE based AL would be the most hydrophobic layer, with the lowest H_2_O absorption. On the contrary, this PTFE based AL absorbed much more water than the VITO CaSE™ AL (based on PSf). The explanation for this result is related to the amount of uncovered hydrophilic C during the manufacturing of an AL, a certain amount of active carbon (S_BET_: 745 m^2^·g^−1^) is covered by the polymer. An AL with a high inner surface area is thus an indication that a large amount of this hydrophilic C remains uncovered and available to absorb H_2_O.

Another advantage of the VITO CoRE™ AL is its electric resistance, being notably lower than that of the VITO CaSE™ electrode (Table 1). This can be explained by the fact that the amount of connections between the electrically conducting carbonaceous particles increases with the share of uncovered carbon in the AL. The pore diameters listed in Table 1 are the mean flow pore diameters as determined with capillary flow porosimetry, i.e., continuous pores connecting both surfaces of the active layer. As a result of the very high pressures applied during the manufacturing process, the ALs fabricated by cold rolling (based on PTFE as a binder) feature an average pore diameter about one order of magnitude smaller than that of the active layer fabricated by casting.

#### 2.1.2. Effect of Composition of the Coagulation Bath on the Properties of the Active Layer 

A comparative evaluation of the effect of the composition of the coagulation bath on the porosity of the active layer was performed for ALs composed of 70 wt % active carbon powder (Norit^®^SX-1G) +30 wt % of PSf (12 wt % in NEP) as the binding polymer. As already discussed, the properties and structure of the porous active layer can be affected by the kinetics of the solvent extraction process during which the AL of the electrode is being formed. Amongst other things, the extraction kinetics is dependent on the composition of the non-solvent. The effect of two non-solvents was investigated: pure water for a fast extraction and a mixture of 75 wt % NMP + 25 wt % water for a slow extraction. These results are shown in Table 2.

The most striking result observed was that using a solvent/non-solvent mixture in the coagulation bath hardly affected the hydrophilic porosity; for ALs with the same composition, the hydrophilic pore volume (ε_hydrophilic_) was very low when compared with the total porosity, as calculated from the iso-propanol absorption (ε_TOTAL_). The higher specific surface area, as obtained with the fast H_2_O extraction, is an indication that here more active carbon (and thus electrochemically reactive sites) is available for the redox reactions to occur. The other characteristics are not quite dependent on the type of extraction bath that is being used.

#### 2.1.3. Effect of the Type of Activated Carbon on the Properties of the Active Layer 

**Figure 2:**
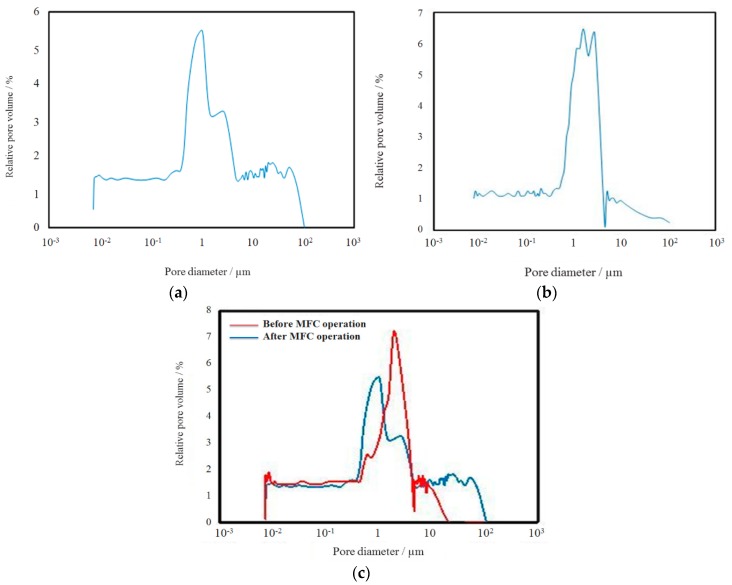
(**a**) Pore size distribution of a HGDL with composition FEP:PSf 75:25; (**b**) Pore size distribution of an AL/SS without HGDL (determined by Hg intrusion porometry); (**c**) Effect of MFC operation on pore size diameter.

The properties of active layers as a function of the type of carbonaceous active powder and the composition of the coagulation bath were studied and are presented in Table 2. The specific surface area of the Norit^®^SX-1G type of C is as high as 745 m^2^·g^−1^ with 50 mass-percent of the particles smaller than 25 µm. For the sake of comparison, Printex^®^25 an alternative type of carbonaceous active powder with a specific surface area (45 m²·g^−1^) was investigated along with Norit^®^SX1G. The highest specific surface areas were obtained with the extractions in water and the highest wettability (expressed as ε_hydrophilic_) was observed for the layers based on Norit^®^SX1G. Among these, the layer prepared using water as coagulation bath was selected for further investigation. Figure 2a shows the pore size distribution of an AL based on Norit^®^SX1G as active powder and a pure water coagulation bath. The highest relative pore volume is situated at pore diameter ca. 1 µm.

#### 2.1.4. Effect of Active Carbon Contents on the Properties of the AL

Three different ratios C:PSf were explored, using pure water as coagulation bath (Table 3). The results clearly show a decrease of the resistance of the AL on increasing the wt % C contents. This is expected because the amount of connections between the electrically conducting carbon particles increases with the amount of carbonaceous powder present in the AL. Other properties such as porosity, specific surface area (S_BET_) or the water absorption coefficient (A) did not vary significantly as a function of wt % C content within the composition span investigated.

### 2.2. Physico-Chemical Characterization of the Hydrophobic Gas Diffusion Layer (HGDL)

The ability of the HGDL to prevent electrolyte leakage was evaluated ex-situ. The results are presented in Table 4. The layers tested in these experiments had two free surfaces instead of normally one. Because of the phase inversion manufacturing procedure a cast porous layer always exhibits two different surfaces: a surface with an open porosity, in contact with the support, and the top surface with a dense porosity [14,15,16,17,18,19,20], Figure 1b. During the water permeability experiments, extremely small amounts of water could penetrate inside the different structures but the dense layer at the low pressure side was the ultimate water barrier for every single HGDL. The water absorption was almost independent of the composition of the HGDLs and was as low as about 3 µL·cm^−2^·h^−^·Pa^−1^, even for the HGDL based on pure PSf.

A series of freestanding HGDLs with a systematic variation in the ratio of hydrophobic particles to PSf was produced and tested. Table 4 shows that the hydrophilic character decreased with increasing amounts of hydrophobic polymer particles. The lower the permeability values, the lower the leakage or amount of weeping of electrolyte that can be expected as a function of time. The HGDL containing 10 wt % FEP showed slightly higher ε_hydrophilic_, than that containing 10 wt % PTFE but it was preferred for its superior processability. Compared with the pure PSf HGDL, the addition of the hydrophobic polymer particles resulted in a considerable improvement in the efficiency of the HGDL (in terms of impermeability to electrolyte), which was confirmed during in-situ testing in electrochemical cells.

Figure 1a shows the surface of the hydrophobic layer. The section on the right shows that, just beneath the surface, small stretched pores are present. On average these slit shaped pores have a diameter of only about 1 µm. Going deeper into the structure the picture shows the hydrophobic FEP particles as they are dispersed in the PSf matrix. The pore size distribution is shown in Figure 2b and confirms the average surface pore size distribution as observed in Figure 1b. The total porosity of the HGDLs, as measured with isopropanol, is almost independent of the amount of hydrophobic polymer in the PSf layer and is situated at a level of ca. 70 ± 10 vol % of the layer. This value enables a sufficient flow for the air to access the active layer of the electrode.

### 2.3. Electrode Characterization in Cathodic Half-Cell Environment

#### 2.3.1. Electrode Polarization

The O_2_ reduction reaction on the cathode half cell set-up proceeds as follows:

pH < 7

O_2_ + 4 H^+^ + 4 e^−^ → 2 H_2_O (1)

pH > 7

O_2_ + 2 H_2_O + 4 e^−^ → 4 OH^−^ (2)

with E = 1.007–0.05916 pH vs. Ag/AgCl at 25 °C and P_O2_ = 0.1

O_2_ reduction reaction can also lead to the synthesis of hydrogen peroxide:

pH < 7

O_2_ + 2 H^+^ + 2 e^−^ → H_2_O_2_ (3)

pH > 7

O_2_ + H_2_O + 2 e^−^ → HO_2_^−^ + OH^−^ (4)

For 2e^−^ reduction half- reactions E are 700 mV and −65 mV vs. SHE [24] (i.e., 500 mV and –260 mV vs. Ag/AgCl) respectively. Their equilibrium is also pH dependent. Subsequent reduction of H_2_O_2_ into water can also take place.

pH < 7

H_2_O_2_ + 2 H^+^ + 2e^−^ → H_2_O (5)

pH > 7

HO_2_^−^ + H_2_O + 2 e^−^ → 3 OH^−^ (6)

**Figure 3:**
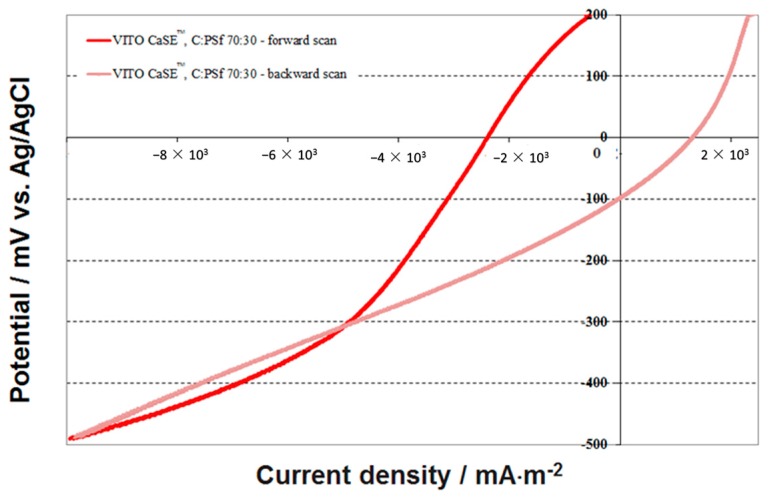
LSV traces for a VITO CaSE™ electrode (C:PSf 70:30), forward and backward scan (scan rate: 1 mV·s^−1^).

E for reactions (5) and (6) are 1760 mV and 870 mV vs. SHE [28] (i.e., 1560 mV and 870 mV vs. Ag/AgCl) respectively. An important issue which is often neglected in LSV experiments is the hysteresis and the complex shape of the voltammogram as shown in Figure 3.

This could be an indication that the system is not in equilibrium and that the results have only some indicative value. Also, the extent of the hysteresis can be related to the degree of adsorption, if the latter is a relevant step in the electrolytic mechanism as is the case for the O_2_ reduction reactions in porous carbon electrodes. The forward scan should be related to the reduction of O_2_ which is present in the electrolyte. It must be clear from half-reactions (1) and (2) that the O_2_ reduction potential is highly dependent on the pH of the electrolyte which in turn is dependent on the amount of charge flowing through the electrode; by the end of the forward scan the pH may have shifted by one unit; the same applies for half-reactions (3) and (5). It is also clear that both the pH and the resulting ionic resistance inside the very small pores of the electrode are not instantaneously at the level of the buffered and circulating bulk electrolyte. The same holds for the O_2_ concentration inside the pores because the O_2_ transport through both the HGDL and AL is too slow to compensate for the reduced amount of O_2_. Additionally the hydrophilic and/or hydrophobic groups on the pore surface may also affect the transport of the reacting compounds to the internal pores. The overall result is that during the scan, both the reversible potential and the polarization are continually changing; as a result, a pronounced hysteresis becomes inevitable, even after selecting an acceptably low scan rate as 1 mV·s^−1^.

By the end of the backward scan the current becomes even positive at cathode potential higher than −100 mV vs. Ag/AgCl. This could be a further indication that during the forward scan, in parallel with the O_2_ reduction reaction to H_2_O some other reduction reaction (e.g., O_2_ reduction to H_2_O_2_) has taken place, the product being re-oxidised at the higher potential. Redox reactions taking place at the functional groups of activated carbon may also contribute to this phenomenon to a minor extent.

The effect of the operational conditions has been determined both on the electrochemical performance and on the physico-chemical properties of the AL. The tested VITO CaSE™ electrode was composed of a current collector, an AL (70 wt % Norit and 30 wt % PSf), but no HGDL. For this experiment no HGDL was applied because it is not possible to determine the physico-chemical properties of an active layer if it is covered with a HGDL. After 14 days the cell was dismantled to evaluate the physicochemical parameters of the electrode and compare them with those of a fresh electrode. The most remarkable result is that only 14 vol % of a fresh electrode can be filled with water whereas after operation in the MFC this volume is as high as about 44 vol %. This is a clear indication that the electrode has become much more hydrophilic, probably related to the evolution of carbon–oxygen or carbon–ORR–adsorbed intermediates. These may create additional vacancies and, combined with the adsorption of impurities inside the pores of the electrode, lead to the formation of stable defect complexes in which water can be further captured. Comparison of the pore size distribution of the electrode before and after MFC operation (Figure 2c) shows that the relative volume of the first peak is reduced in favor of the relative volume of the second peak. This could indicate that the small pores are involved in the complexation of oxygen and carbon vacancies, which is likely the origin of the increased hydrophilicity of the electrode.

**Figure 4:**
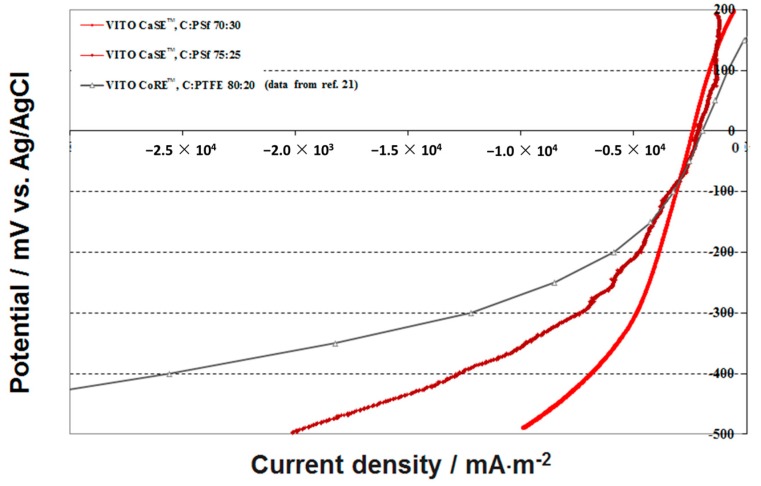
Effect of the composition of the AL (C wt %) on the performance of the VITO CaSE™ electrode.

Figure 4 presents the LSV traces registered for two VITO CaSE™ electrodes. For comparison purposes, results on the VITO CoRE™ electrode from reference [29] are presented as well.

At cathode potentials −100 mV vs. Ag/AgCl all three electrodes produce a similar current density. As is discussed in Subsection 3.4, this region of cathode potential corresponds with the cell operating point leading to maximum power density. The amount of C in the VITO CaSE™ electrodes becomes only relevant at potential lower than −500 mV. It is also in that region that the calendered electrodes show superior performance compared to that of the VITO CaSE™ electrodes. It should be noted nonetheless that these current densities are on average ca. 10 times higher than the corresponding values from the long term tests (discussed below), which are performed at a constant electrode potential of −100 mV vs. Ag/AgCl.

#### 2.3.2. Electrode Performance as a Function of Time

**Figure 5:**
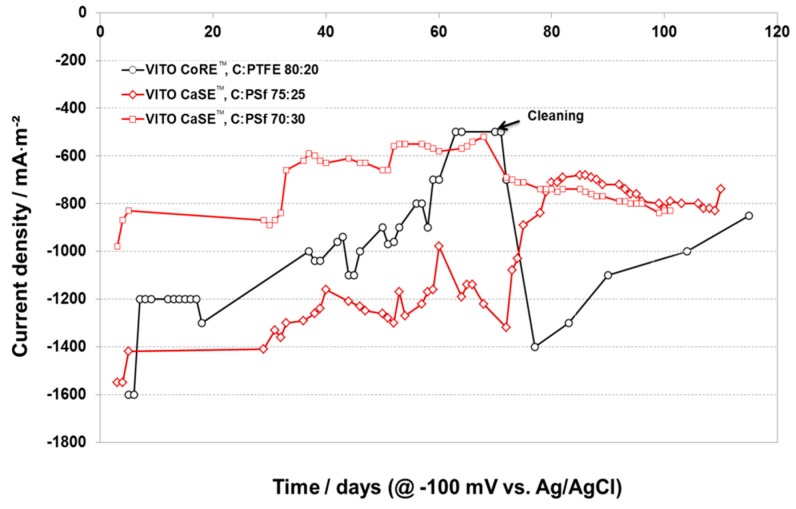
Performance of a VITO CaSE™ electrode (C:PSf 70:30) as a function of time at constant voltage −100 mV vs. Ag/AgCl.

Two different compositions of VITO CaSE™ electrodes (C:PSf 75:25 and 70:30 in the AL respectively) and one VITO CoRE™ (C:PTFE 80:20 in the AL) electrode (C:PTFE 80:20 in the AL) were tested in continuous operation at constant potential for more than 100 days. Figure 5 clearly shows that both the VITO CaSE™ and VITO CoRE™ electrodes suffer from a quite severe performance loss during the first hours of operation. This is probably related to the equilibrium settings of the electro-active species inside the porous structure of the AL. During the first days of operation, the VITO CoRE™ electrode had a performance at a level of about −1200 mA·m^−2^. This value of current density is in between the average performance of the VITO CaSE™ electrode with AL C:PSf 70:30 (ca. −800 mA·m^−2^) and that of the VITO CaSE™ electrode with AL C:PSf 75:25 (ca. −1400 mA·m^−2^). However, the current density obtained with the VITO CoRE™ electrode evolves towards the performance level of the worst performing VITO CaSE™ electrode (AL C:PSf 70:30); after e.g., 50 days in operation the current densities for the VITO CoRE™ electrode and for the VITO CaSE™ electrodes with AL C:PSf 75:25 and AL C:PSf 70:30 reached −900 mA·m^−2^, −1260 mA·m^−2^ and −660 mA·m^−2^ respectively .The steady decrease in performance was attributed to the adsorption of fouling in the pores of the electrodes, which takes place more readily in the VITO CoRE™ electrode than in the VITO CaSE™ electrode because the latter has a larger pore size. The nature of such deposits could be inorganic (e.g., salts coming from PBS), organic (originating from acetate), biologic (from inoculum) or a mixture thereof. A cleaning experiment was set up to verify this hypothesis. After ca. 2 months of operation the electrodes were cleaned by flushing the cells with demineralized water, unmounting the electrode from the cell, and cleaning them with fresh demi water. The electrodes were subsequently built and tested in the cell again. No clear effect could be observed for the VITO CaSE™ electrodes. In the case of the VITO CoRE™ electrode we concluded that fouling deposits were probably removed with the cleaning experiment, as its performance reverted to its initial level.

### 2.4. Electrode Performance in MFC (Full Cell Configuration)

#### 2.4.1. Power Output and Half-Cell Potentials

**Figure 6:**
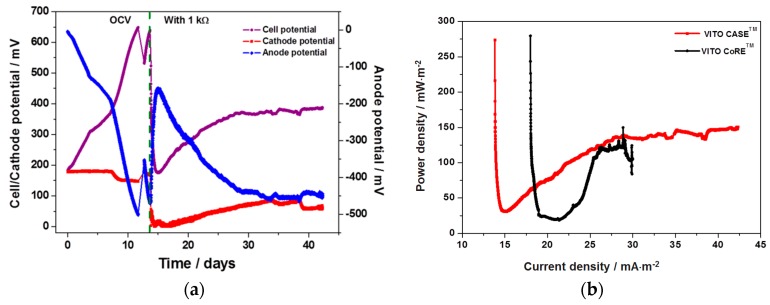
(**a**) Cell potential and anodic, cathodic half-cell potentials against time during MFC operation with VITO CaSE™ electrode as cathode; (**b**) Comparative power density profiles against time during MFC operation with VITO CoRE™ and VITO CaSE™ cathodes.

Anodic and cathodic half-cell potentials along with cell potential and current densities were measured during MFC operation in open circuit and closed circuit modes (Figure 6a). Anode potential should be as low and the cathode potential as high as possible for the maximized electrical energy output. This suggests the need of optimal anode and cathode potentials satisfying both the cell growth and energy output [30,31]. Immediately after start-up the cathode (VITO CaSE^TM^) potential was about 190 mV and was stable till the eighth day of operation where it showed a small drop to 145 mV and stabilized until the circuit was closed. On the other hand, the value of anode potential was almost 0 mV at the start. The value of anode potential gradually decreased until it reached –500 mV at the twelth day of operation, indicating a strong acetate oxidizing environment at the anode. As a result of this change in anode potential, the cell potential increased gradually from 190 mV of start-up potential to 654 mV of maximum performance. A sudden drop in fuel cell performance was observed after the twelfth day due to the depletion of substrate. After addition of substrate, all three potentials gradually recovered the maximum value in two days.

The cathode potential also showed a small drop with respect to the drop in anode potential but increased immediately after substrate addition. This shows the dependence of cathodic reduction on the anodic oxidation reaction. The MFC was then connected in closed circuit using 1 kΩ resistance to measure the current flow across the circuit (Figure 6a). Immediately after connecting in the circuit, within a few hours, the anode potential increased to −150 mV and the respective cathode potential dropped to near 0 mV, contributing to a drop in cell potential of up to 162 mV. Parallel to this, a current discharge was also observed in the circuit immediately after circuit closure (535 mA·m^−2^), which led to a power density of 274 mW·m^−2^ (Figure 6b). The high power density observed immediately after connecting the circuit is due to capacitive current rather than to microbial activities. The current density rapidly dropped to 164 mA·m^−2^ within hours and then started to increase slowly, reaching a stable value at the 30th day of operation (405 mA·m^−2^ to 435 mA·m^−2^), contributing to a stable power output (130 mW·m^−2^ to 150 mW·m^−2^), till the end of operation. After the open circuit phase, the circuit was closed by means of applying a load of 1 kΩ causing the cathode potential to change notably in response to the current discharge. Immediately after connecting in the circuit, the cathode potential dropped to near zero and gradually increased until reaching the steady state potential of 88 mV to 96 mV at the 34th day of operation and was almost stable till the end of operation. The cathode potential took slightly more time to stabilize than the anode potential and current output. In a similar MFC set-up and operation conditions with the VITO CoRE™ electrode as cathode, an almost similar power output was achieved (135 mW·m^−2^ to 145 mW·m^−2^), as recently reported by our group [26]. However, the cell potential was slightly higher compared to the CaSE™ operation; in this respect it should be noted that the VITO CoRE™ electrode has a higher carbon percentage in the AL than either of the VITO CaSE™ electrodes evaluated. The cathode potential was observed to be stable both with the VITO CaSE™ electrode and the VITO CoRE™ electrode, suggesting that it is feasible to keep the cathode potential independent of anode functioning, as is expected for a single chamber MFC.

#### 2.4.2. Fuel Cell Characterization

**Figure 7:**
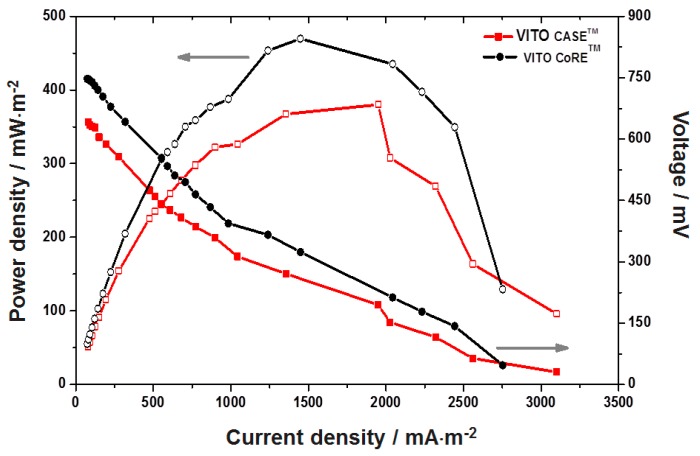
Comparative polarization profiles across varying external loads (10 kΩ–10 Ω) during MFC operation with VITO CoRE™ and VITO CaSE™ cathodes.

Once the power output was stabilized, polarization characteristics were recorded to evaluate the fuel cell behavior. After stabilization at 1 kΩ load, MFC was evaluated for its performance under varying external loads (10 kΩ–10 Ω). High external resistance solely limits the electron delivery to the cathode, while, at lower external resistance, the electron delivery to the cathode is limited by kinetic and/or mass transfer (or internal resistance). At the point where both the external and internal resistances become equalized, the power density shows a peak value, which is commonly the cell design point (CDP) of choice for MFCs [31]. Typical polarization behavior was observed with a gradual increment in power density with decreasing resistance up to (CDP) and then decreased afterwards with both the cathodes (Figure 7). Electron discharge from the biocatalyst to the anode is regulated by the external and internal resistances [32].

The voltage drop at higher resistances was slow, stabilized faster, and regained the original voltage quicker after removing the load, while it was reverse at lower resistances. The voltage drop and electron discharge started visibly at 10 kΩ (68 mA·m^−2^) and gradually increased to 3100 mA·m^−2^ at 10 Ω, accounting for a voltage drop of 31 mV. The CDP was observed at 100 Ω with a voltage of 195 mV and current density of 1950 mA·m^−2^, accounting for a power density of 380 mW·m^−2^. It is usual practice to operate fuel cells beyond CDP because the fuel cell will destabilize at higher current densities and low voltages. Similarly, the MFC operation with VITO CoRE™ also showed a typical polarization behavior depicting an increment in current densities associated with voltage drop, as the resistance decreases. Electron discharge started visibly at 7 kΩ (105 mA·m^−2^) and increased with lowering resistance till 10 Ω (2750 mA·m^−2^). The power density obtained with the VITO CoRE™ electrode increased when increasing the external resistance to reach a maximum when 200 Ω was applied (1450 mA·m^−2^; 469 mW·m^−2^) and dropped thereafter. Though, the current density is lower than VITO CaSE™ MFC, the power density was higher in this case, which is due to the regulation in voltage drop. The percentage of carbon in the AL is higher (80%) in the VITO CoRE™ electrode than in the VITO CaSE™ electrode (70%), which allows the electrode to maintain a higher potential difference resulting in higher power density. However, the higher porosity in the gas diffusion layer of the VITO CaSE™ electrode allowed better gas diffusion. This ultimately leads to a higher reaction rate at the interface, resulting in slightly higher current densities.

## 3. Materials and Methods 

### 3.1. Materials

Activated carbon powder was obtained from commercial sources Norit SX-1G (Norit B.V., Amersfoort, The Netherlands) and Printex 25 (Evonik Industries, Essen, Germany). Polysulfone (PSf, UDEL^®^ P-1800 NT 11) was supplied by Solvay (Brussels, Belgium). PTFE Algoflon^®^ was supplied by Solvay Solexis Spa (Bollate, Italy). Fluorinated ethylene-propylene copolymer (FEP 8000) and PTFE (type 636N) were purchased from DuPont de Nemours (Nederland) B.V. (Dordrecht, The Netherlands). Stainless steel (SS) 316L gauze (Solana, Schoten, Belgium) wire diameter 100 µm, mesh 44 was used as current collector.

### 3.2. Manufacturing of the Gas Diffusion Cathodes

The manufacturing of the complete VITO CaSE™ electrode comprises the steps of casting the AL onto the current collector and casting a hydrophobic gas diffusion layer HGDL onto the active layer. The process is described below for each of these two steps. For comparison purposes some results on the characterization of electrodes prepared by cold rolling (VITO CoRE™ electrodes, VITO, Mol, Belgium) are presented as well. These electrodes are based on activated carbon and PTFE (type 636N, DuPont) .The performance of these electrodes has already been described in detail earlier for various systems [10,24,25,26,27,33,34].

#### 3.2.1. Active Layer (AL)

For the manufacturing of the AL, a solution of polysulfone in N-ethyl-pyrrolidone (NEP) is mixed with activated carbon powder. A typical active layer for MFC application is composed of 70 wt % active carbon and 30 wt % PSf. On the lab scale 48 g PSf was dissolved in 352 g of NEP, after which the PSf solution was mixed with 112 g of activated carbon. The suspension was subsequently deaerated at room temperature under static vacuum (50 mbara). The casting head was filled with the suspension which was then cast onto the support by means of longitudinal motion of the casting head. The wet active layer (supported with the current collector net) was next immersed in the coagulation bath. The active layer formed in the coagulation step was boiled in demineralized water to remove the residual solvent and non-solvent and dried overnight at room temperature.

#### 3.2.2. Hydrophobic Gas Diffusion Layer (HGDL)

For the HGDL, hydrophobic polymer particles were added to a solution of 15 wt % PSf in NEP. The hydrophobic particles were FEP 8000, PTFE Algoflon or PTFE 636N. A typical HGDL is composed of 75 wt % of a hydrophobic material and 25 wt % PSf. The suspension to produce such HGDL is typically composed of 60 g PSf dissolved in 340 g NEP and 180 g of the hydrophobic polymer particles. For the rest, the same manufacturing procedure was used as described for the AL except that in this case the HGDL was cast onto the AL of the electrode.

### 3.3. Manufacturing of Freestanding Layers

Freestanding specimens of AL and of HGDL were manufactured and characterized for the screening of optimal compositions. These layers were prepared by casting on a glass plate as a temporary support, subjecting the cast layer to phase inversion by immersing in a coagulation bath and removing residual solvent by treating the layer in hot demi water over 1 h after separating the layers from their temporary support. When PSf was the only component for the HGDL, the water repellent performance was not satisfactory due to their relatively low hydrophobicity, resulting from the high surface area of PSf. In a second stage different amounts of hydrophobic polymer powders were added to the polysulfone solution in order to increase the hydrophobicity of the resulting layer. A series of different PSf based HGDLs incorporating increasing amounts of different hydrophobic polymer particles (from 5 up to 80 wt %) were manufactured and characterized. The thickness of the layers was measured with a Mitutoyo micrometer (Kawasaki, Japan). The thickness was measured at four different points in order to obtain a representative value. The freestanding ALs typically had a final thickness of ca. 700 µm. The freestanding HGDLs had a final thickness of ca. 100 µm.

### 3.4. Physicochemical Characterization Techniques

The procedure for the determination of hydrophilic porosity (ε_hydrophilic_, %) and total porosity, ε_TOTAL_, %), and of the electric resistance of the free standing active layers has been described elsewhere [27]. Pore size distribution was determined by Hg intrusion porosimetry with a Pascal 240 Porosimeter (Thermo Electron Corporation, Waltham, MA, USA). Pore diameters were also determined by capillary flow porometry (CFP) using a PMI Capillary Flow Porometer 6.0 (Porous Materials Inc., New York, NY, USA). With the latter technique only the open pores going from one side of the layer to the other are measured.

The absorption coefficient (Aw, mg cm^−2^·s^−1/2^) was calculated for freestanding ALs from the amount of water absorbed by capillarity per surface unit (mg·cm^−2^), as per Equation 7:

(7) $$A_{w}=\frac{m_{H2O,t}}{S\cdot t^{1/2}}$$

where t is the time (s), m_H2O,t_ (g) is the mass of water absorbed by the sample after time t of immersion and S (cm^2^) is the geometric area of the sample in contact with water.

The pore volume and the BET surface area (S_BET_) were obtained from N_2_ adsorption experiments (Nova 3000, Quantachrome, Boynton Beach, FL, USA), using Quantachrome NovaWin 2 (Version 2.2) for processing the data. Typical measuring protocol comprises degassing for 20 h at 120 °C, and subsequent measurement of the adsorption isotherm at −196 °C. Field emission scanning electron microscopy (FESEM) and EDS spectra were performed using a Jeol JSM-6340F (Peabody, MA, USA).

An in-house set up was used for the determination of water transport through the HGDL. The dry HGDLs were incorporated in a horizontal position into a Plexiglas cell (projected surface area 13.85 cm^2^), with the dense porosity at the low pressure side. A plastic tube on top of the cell was filled with water to apply a pressure difference across the dry HGDL sample. A typical experiment would proceed continuously over 3–5 days at room temperature. The transmembrane volumetric flux was determined from the very small reduction of the water level in the plastic tube on top of the cell (no trace of water could be observed at the bottom of the cell, underneath the HGDL sample). The water permeability Lp is defined as the volume of water that passes through a membrane per unit time, per unit area, and per unit of transmembrane pressure (8).

(8) $$L_{p\;}=\frac{J}{\Delta p}$$

It was calculated by dividing the flux by the pressure at which it was measured, considering that 1 m water column accounts for 10 KPa.

### 3.5. Electrode Characterization in Cathodic Half-Cell Configuration

**Figure 8:**
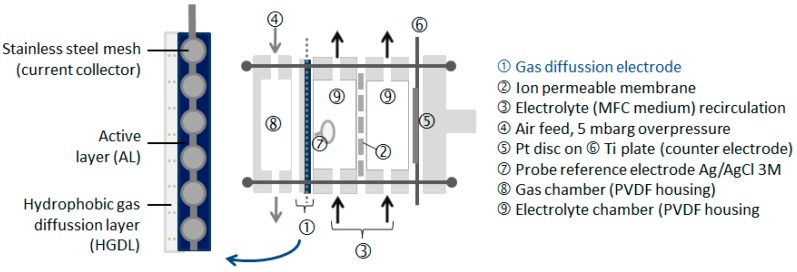
Schematic representation of the cathode half-cell and cross section of the electrode (inset).

Characterization of the electrodes as oxygen (air) reducing cathode was carried out in an electrochemical half-cell under conditions analogous to those described in previous work [21]. Figure 8 is a schematic reresentation (side view) of the cathode half-cell used for the characterization of the electrodes. The projected surface area of the electrode was 10 cm^2^. The experiments were carried out at room temperature (18 °C). Air was fed at an overpressure of 5 mbarg. All values of electrode potential are reported versus Ag/AgCl (3 M KCl) reference electrode (+199 mV vs. SHE). Electrochemical measurements were performed using a potentiostat type VersaSTAT 3F (Princeton Applied Research, Oak Ridge, TN, USA) and the VersaStudio software (Princeton Applied Research, Oak Ridge, TN, USA) for data collection. Unless otherwise stated all the Linear Sweep Voltammetric (LSV) experiments were performed at a scan rate of 1 mV·s^−1^. The electrode potentials were always scanned from +200 mV to −500 mV vs. Ag/AgCl (3 M KCl). The curves are shown for the forward scan unless otherwise noted.

For the investigation of the evolution of performance with time, the cathode potential was fixed at −100 mV vs. Ag/AgCl and the cathodic current was monitored on a daily basis. VITO CaSE™ electrodes (FEP:PSf 75:25 in the HGDL; C:PSf 75:25 and 70:30 respectively in the AL) and one VITO CoRE™ were tested under identical conditions for comparison purposes.

### 3.6. Electrode Characterization in MFC (Full Cell Configuration)

Full MFC was constructed as described in our previous experiments [26], using a carbon felt anode and an enriched mixed culture as source of inoculum, with a VITO CaSE™ electrode as cathode. The projected surface area of both the electrode was 10 cm^2^ and the anodic volume was about 30 mL. The experiments were carried out at room temperature (18 °C) in a batch re-circulated mode with a feed bottle of 800 mL working volume. Air was fed at an overpressure of 5 mbarg over a water column at the cathode side and the feed bottle was continuously sparged with N_2_ to create an anaerobic environment at the anode. Initially, MFC was operated in open circuit mode till a stable voltage was observed and then connected across 1 kΩ resistance to measure the current and power densities. Once the MFC was stabilized in closed circuit, the performance of the MFC was evaluated in terms of polarization. Polarization studies were carried out under a varying external load from 10 kΩ to 10 Ω, measuring the voltage drop and current manually using a digital multi-meter. The respective current and power densities were calculated with respect to the anode surface area and plotted. Shifts in both the anode and cathode potentials were continuously monitored throughout the experiment.

## 4. Conclusions

VITO has developed an electrode manufacturing process based on film casting and phase inversion, which would enable the production of large multilayered electrodes by a continuous manufacturing process. Physico-chemical properties of the electrode’s active layers such as total porosity, hydrophilic porosity, and BET surface area maintain a direct relation with their composition (carbonaceous powder and binder used, ratios of carbon) and the fabrication method. The electrodes have been evaluated for application in BESs and compared to electrodes fabricated by cold rolling. A few long duration tests in the cathode half-cell emulating MFC environment demonstrated that the VITO CaSE™ electrode is more stable than the VITO CoRE™ electrode. In a full MFC mode, the VITO CaSE™ electrode showed higher current densities, while the VITO CoRE™ electrode showed higher power densities. This is due to the differences in carbon percentage in the AL and the porosity of the GDL. The hydrophobicity of the VITO CaSE™ AL should be further reduced to the level of the VITO CoRE™ AL. This is maybe possible by further increasing the amount of C in the active layer. For final improvement, the fine tuning of the hydrophobic and hydrophilic capillaries will be necessary. The possibility of casting the AL in one run with the HGDL is under investigation. Material choice and parameters used for the casting process can be adapted in order to produce gas diffusion electrodes tailored for other electrochemical applications.

## Figures and Tables

**Table 1:** Influence of polymer binder and fabrication method on the properties of the active layer (composition 80 wt % C:20 wt % polymer).

Polymer	Surface Energy (Polymer), mN·m^−1^	Thickness, cm	Resistance, Ω·cm	ε_hydrophilic_, %	ε_TOTAL_, %	S_BET_, m^2^·g^−1^	Pore Diameter, µm
PSf	41	0.0357	194	10	65	230	0.5
PTFE	20	0.0510	14	29	66	454	0.1

**Table 2:** Influence of the composition of the coagulation bath and of the type of active carbon used on the properties of the cast active layer (composition 70 wt % C:30 wt % PSf).

Non-Solvent	Type of Carbon Powder	Thickness, cm	Resistance, Ω·cm	Aw, mg·cm^−2^·s^−1/2^	ε_hydrophilic_, %	ε_TOTAL_, %	S_BET_, m^2^·g^−1^
H_2_O	Norit SX1G	0.076	145	0.27	8	74	207
H_2_O	Printex	0.068	321	–	1	72	18
NMP/H_2_O	Norit SX1G	0.070	99	0.34	9	73	172
NMP/H_2_O	Printex	0.066	nm *	–	0.5	65	15

* sample broken.

**Table 3:** Effect of wt % carbon on the properties of the cast active layer (AL).

C, wt %	Thickness, cm	Resistance, Ω·cm	Aw, mg cm^−2^ s^−1/2^	ε_hydrophilic_, %	ε_TOTAL_, %	S_BET_, m²·g^−1^
65	0.071	655	0.42	8	73	177
70	0.076	145	0.27	8	74	207
75	0.067	86	0.33	10	70	201

**Table 4:** Effect of the additional polymers on the properties of the HGDL.

Polymer Additive	wt %	Thickness, cm	Mean Pore Size (CFP), μm	ε_hydrophilic_, %	ε_TOTAL_, %	L_P_, L·h^−1^·cm^−2^·bar^−1^·10^6^
FEP	0	0.091	0.405	32	79	3.6
FEP	10	0.097	0.162	28	78	3.7
FEP	20	0.096	0.202	27	81	1.7
FEP	40	0.106	0.115	15	75	2.8
FEP	60	0.103	0.159	7	74	2.5
FEP	70	0.107	0.116	4	73	2.8
FEP	80	0.117	0.136	3	66	4.2
PTFE Algoflon	10	96	0.358	21	80	3.7
PTFE 636N	10	0.105	0.137	24	63	4.1

## Abbreviations

The following abbreviations are used in this manuscript:

AL	(electrochemically) active layer
A_w_	Absorption coefficient
CDP	cell design point
CFP	capillary flow porometry
FEP	Fluorinated ethylene propylene
GDE	Gas diffusion electrode
HGDL	hydrophobic gas diffusion layer
L_P_	Liquid Permeability
LSV	Linear Sweep Voltammetry
MES	Microbial Electrochemical System
MFC	Microbial Fuel Cell
NEP	N-Ethylpyrrolidone
ORR	oxygen-reduction reaction
PSf	Polysulfone
PTFE	Polytetrafluoroethylene
S_BET_	BET Specific Surface Area
SEM	Scanning Electron Microscopy
